# 2D Electronic Spectroscopic Techniques for Quantum Technology Applications

**DOI:** 10.1021/acs.jpcc.1c02693

**Published:** 2021-06-11

**Authors:** Elisabetta Collini

**Affiliations:** Department of Chemical Sciences, University of Padova, via Marzolo 1, 35131 Padova, Italy

## Abstract

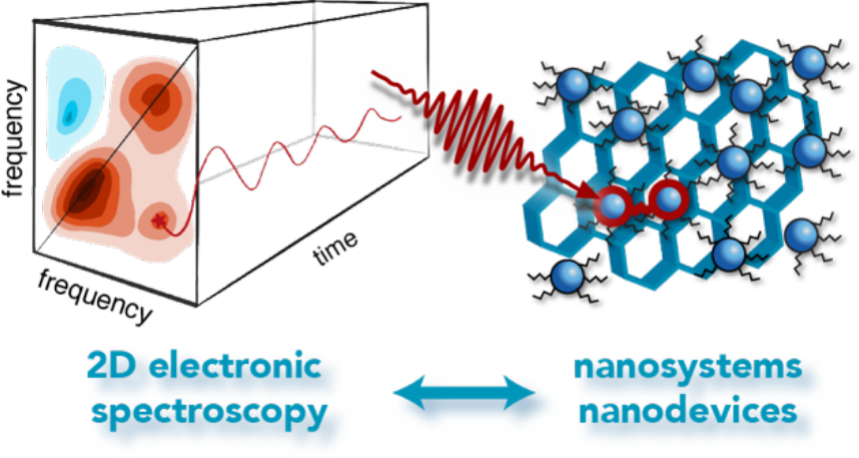

2D electronic spectroscopy (2DES) techniques have gained particular interest given their capability of following ultrafast coherent and noncoherent processes in real-time. Although the fame of 2DES is still majorly linked to the investigation of energy and charge transport in biological light-harvesting complexes, 2DES is now starting to be recognized as a particularly valuable tool for studying transport processes in artificial nanomaterials and nanodevices. Particularly meaningful is the possibility of assessing coherent mechanisms active in the transport of excitation energy in these materials toward possible quantum technology applications. The diverse nature of these new target samples poses significant challenges and calls for a critical rethinking of the technique and its different realizations. With the confluence of promising new applications and rapidly developing technical capabilities, the enormous potential of 2DES techniques to impact the field of nanosystems, quantum technologies, and quantum devices is here delineated.

## 1 Introduction

The ability to spectroscopically probe ultrafast events in the femtoseconds (fs) time regime has been crucial for understanding fundamental scientific questions in biology, chemistry, and physics.^1^ Examples of such investigations include transition-state dynamics of chemical reactions, solute–solvent interactions, energy and charge transfer, excitonic interactions, and quantum coherence, just to cite a few.^2^ Several ultrafast spectroscopy techniques have been developed to this aim, including pump–probe (probably the most famous and widespread among the fs techniques), various types of photon echo, transient grating, resonant coherent Raman, and hole-burning spectroscopies, and optical Kerr spectroscopy. All these techniques can be broadly classified as four-wave mixing (FWM) techniques. In a perturbative approach, the FWM process is governed by the third-order nonlinear response function, the lowest order to access information on the evolution of excited states.^3,4^

Bidimensional electronic spectroscopy (2DES) is an extension of the FWM techniques, and it has several analogies with conventional “monodimensional” (1D) spectroscopies like photon echo and pump–probe. The development of multidimensional techniques represented a remarkable advance in the field of ultrafast spectroscopy because it gave access to a series of subtle observables, usually hidden in typical 1D techniques, which can potentially open up new exciting perspectives in the emerging fields of nano and quantum technologies.

What distinguishes a 2D from a 1D technique is that in 2D techniques, each of three light–matter interactions is carefully controlled in time, and the phase and amplitude of the third-order signal are measured. In this way, the medium’s response can be cast into 2D spectra, which provide more straightforward and direct access to signal contributions hidden within the broad lineshapes of 1D spectra. This allows revealing with improved reliability details on molecular structure, vibrational and electronic motions, interactions, couplings, and relaxation processes. This is particularly advantageous in the investigation of condensed phases, in which complex inter- or intramolecular interactions and environmental heterogeneity may be intertwined within the broad spectral features of 1D measurements. 2D techniques instead permit the spreading of congested spectra along multiple time or frequency coordinates.

At the dawn of 2DES in the early 2000s, the technique was mainly applied to biological light-harvesting antenna complexes and still now its fame is linked to the investigation of such systems.^5,6^ Indeed, 2DES appeared as the ideal technique to investigate the possible presence and the relevance of coherent quantum mechanisms active during the biological light-harvesting processes. In fact, the development of 2DES, now recognized as the primary tool to obtain clear and definitive experimental proof of such effects, has been central in the advent of quantum biology.^7^ Although the effective role of quantum phenomena on the biological light-harvesting process is still a matter of intense debate,^8,9^ inspire by nature, spectroscopists started to move their attention on biomimetic artificial systems, from organic multichromophore systems^10−12^ to fully inorganic^13−16^ and hybrid materials^17−19^ and to functioning solid-state devices.^20−22^ Based on this wealth of evidence, the enormous potential of 2DES techniques to impact the field of nanosystems, semiconductors, quantum technologies, and quantum devices must now be recognized. Nonetheless, the diverse nature of these new target samples poses important challenges and calls for a critical rethinking of the technique and its different realizations.

To this aim, this Perspective, rather than trying to provide an exhaustive overview of technical details (many excellent books and reviews are already available^6,23−28^), has the ambition of facilitating a critical idea of the boundaries of the 2DES techniques and highlighting the game-breaker role that such techniques can play in the emerging field of quantum technologies.

Now, the main assets of 2DES can be summarized in two points:

(1) First, in 2D spectra, the couplings between different states or transitions are mapped as cross-peaks, far from the diagonal region where the remaining relaxation dynamics occur. Cross-peaks are achievable only in multidimensional techniques. They are the “smoking gun” witnessing the presence of interactions and couplings between states. It is also possible to follow their time behavior, assessing the presence of couplings and the associated dynamics.
(2) Second, the technique is sensitive to coherent dynamics and quantum evolution manifested as oscillations of the signal amplitude at specific coordinates of the 2D maps. The study of frequency, amplitude distribution, and dephasing time of such oscillations allows a complete characterization of any coherent dynamics (electronic, vibrational, vibronic, etc.) taking place during the system relaxation.

To have a physical insight into what is determining these capabilities, the main technical aspects of 2DES and the physical origin of the signal outputted by a 2DES experiment will be quickly outlined in Section 2. In Section 3, different experimental schemes and layouts currently available will be described to evaluate for which uses each configuration is more suitable. Finally, Section 4 summarizes a few recent examples of applications on innovative materials that are indicating exciting developments and new perspectives for multidimensional techniques.

## 2 Electronic Transitions in 2DES Spectra: Couplings and Dynamics

### 2.1 How a 2DES Map Is Built

**Figure 1:**
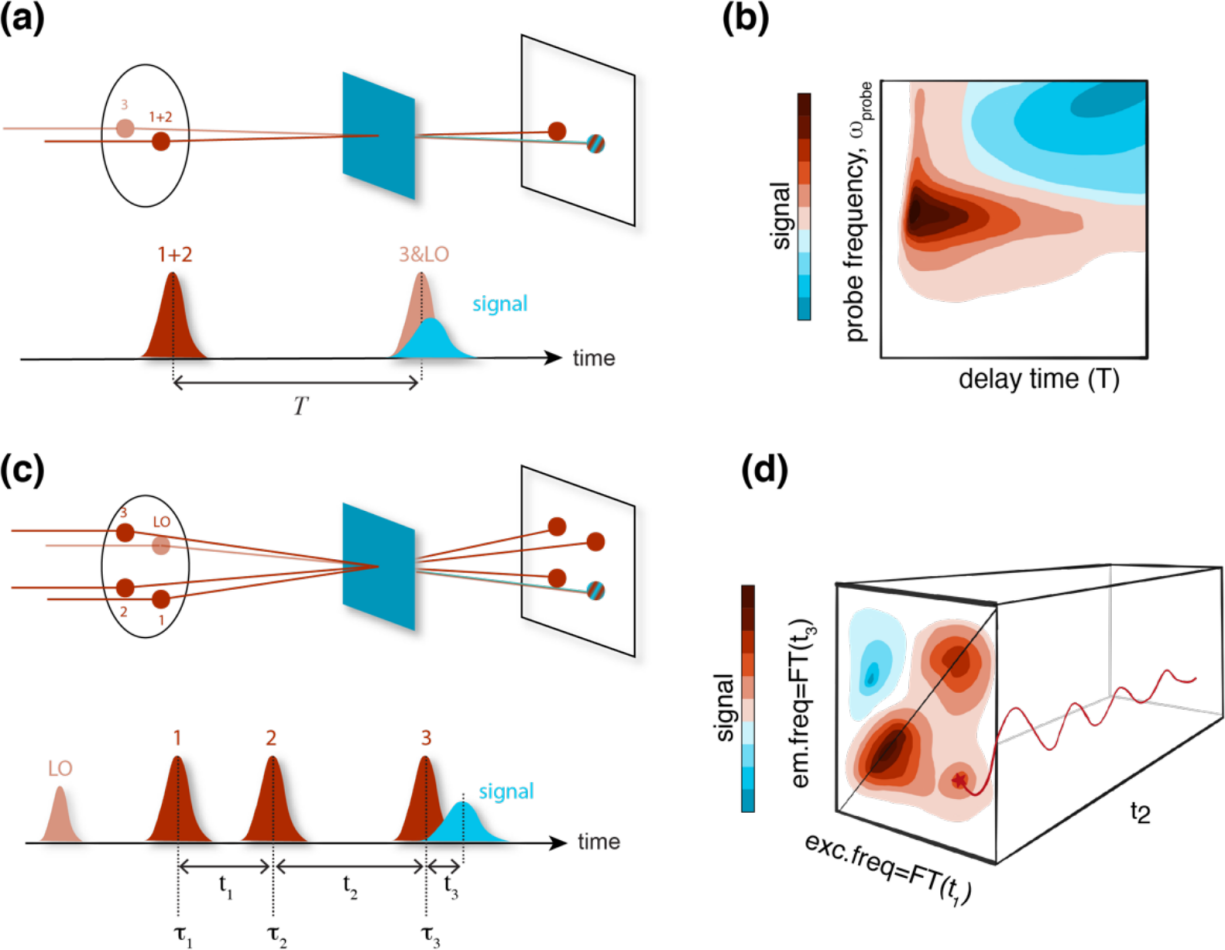
Excitation geometry (upper line) and pulse sequence (lower line) for (a) pump–probe and (c) fully noncolinear 2DES experiments. In the pump–probe experiment, the first two interactions (*E*_1_ and *E*_2_, collectively indicated as the “pump”) are simultaneous and propagate along the same direction. The signal (blue) is emitted in the same direction as the probe (*E*_3_), it is self-heterodyned by it, and it is measured as a function of the delay time *T* between the pump and probe beams. In 2DES experiments the three fields interacting with the sample (*E*_1_, *E*_2_, and *E*_3_) and a fourth beam used only for detection purposes (LO = local oscillator) are arranged at the vertices of a square (BOXCARS geometry. The signal is recorded as a function of the time delays *t*_1_, *t*_2_ and *t*_3_. (b) Example of a typical plot *ω*_*probe*_*vsT* obtained as a result of a pump–probe experiment. (d) Pictorial representation of the matrix data set obtained with a 2DES experiment; the two frequency axes ω_1_ and ω_3_ are obtained by Fourier transforming the delay times *t*_1_ and *t*_3_. The evolution of the 2D (ω_1_, ω_3_) maps is followed along *t*_2_.

To understand how the technique works, a comparison to the more familiar pump–probe technique could be helpful.^24^ In a typical pump–probe experiment, a short pump pulse from a femtosecond laser impulsively excites the sample to an electronic excited state. After a time delay *T*, a weak probe pulse records the changes in the absorption due to the action of the pump (Figure 1a). The transmittance of the probe can be increased (“bleaching” or “stimulated emission”) or decreased (“excited state absorption”) in different spectral regions. Overall, in a pump–probe experiment the signal is plotted as a function of a delay time (*T*) and a frequency (the probe frequency ω_probe_ (Figure 1b)). In a 2D experiment, instead, the signal is plotted as a function of a delay time (population time, *t*_2_) and two frequencies: the axis ω_3_, typically denoted as “emission” frequency, is analogous to the ω_probe_ axis in the pump–probe response. The new frequency axis ω_1_ (“excitation” frequency) can be thought of as the distribution of frequencies excited by the pump pulse. The excitation axis is built in the time domain by scanning the delay *t*_1_ between the first two interactions (Figure 1c). The generated signal oscillates as a function of the delay time *t*_1_, allowing the excitation frequency axis to be recovered by Fourier transform (FT). In this case, the broader the pulse spectrum, the larger the excitation frequency window probed by the experiment is. The result has both high temporal and spectral resolution.

Overall, a 2D map can be interpreted as a frequency–frequency correlation spectrum at a fixed value of the delay time *t*_2_: ***S***^(3)^ (ω_1_, *t*_2_, ω_3_), (Figure 1d). This signal is plotted as a function of ω_1_ (=FT(*t*_1_)), representing the initial excitation, and ω_3_ (=FT(*t*_3_)), which can be interpreted as the ensuing emission.^29,30^

### 2.2 Third-Order Signal in a 2DES Map

The signal plotted in a 2DES map can be formalized in the perturbative approach framework, which allows expressing the total polarization ***P*** as perturbative expansion in powers of the incoming fields. The critical quantity to determine any FWM nonlinear signal (including 2DES signal) is the third-order polarization ***P***^(3)^, to which the signal is proportional. ***P***^(3)^ can be expressed as the convolution of the nonlinear response function ***R***^(3)^(*t*_1_,*t*_2_,*t*_3_) with the pulse fields ***E***_*j*_(*k*_*j*_,*t*):^3,30^
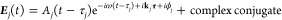
1
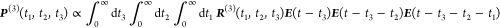
2where the *j*th laser pulse is centered at *τ*_*j*_ and ***k***_*j*_, ω, *A*_*j*_(*t*), and *ϕ*_*i*_ are the wavevector, carrier frequency, temporal envelope and phase of the field. *t*_1_, *t*_2_, and *t*_3_ are the time intervals between interactions, as illustrated in Figure 1c.

**Figure 2:**
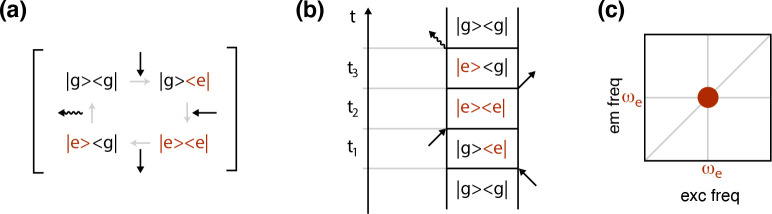
(a) Example of pathway contributing to the time evolution of the density matrix after the interaction with three laser pulses (black arrows). The wavy arrow represents the emitted signal. A two-level system (ground state g and excited state e) is considered. (b) Representation of the same pathway in terms of the Feynman diagram. (c) Signal corresponding to this diagram. This is expected to appear in the 2D map as a diagonal peak at coordinates (ω_e_, ω_e_), with ω_e_ being the frequency of the transition g → e.

The response function *R*^(3)^(*t*_1_,*t*_2_,*t*_3_) can be expressed as the sum of several terms, which correspond to all possible contributions of three incoming fields to the polarization. Each of these terms can be associated with a specific time evolution of the density matrix, known as Liouville pathways. In spectroscopy, these pathways are usually graphically visualized by means of diagrams capable of highlighting the temporal sequence of the field interactions and the transitions promoted in the systems by such interactions. The most famous are the double-sided Feynman diagrams. Figure 2 reports an example of a pathway contributing to the time evolution of the density matrix (Figure 2a), its corresponding representation in terms of a Feynman diagram (Figure 2b) and the coordinates in the 2D map at which the corresponding signal is expected to appear (Figure 2c).

**Figure 3:**
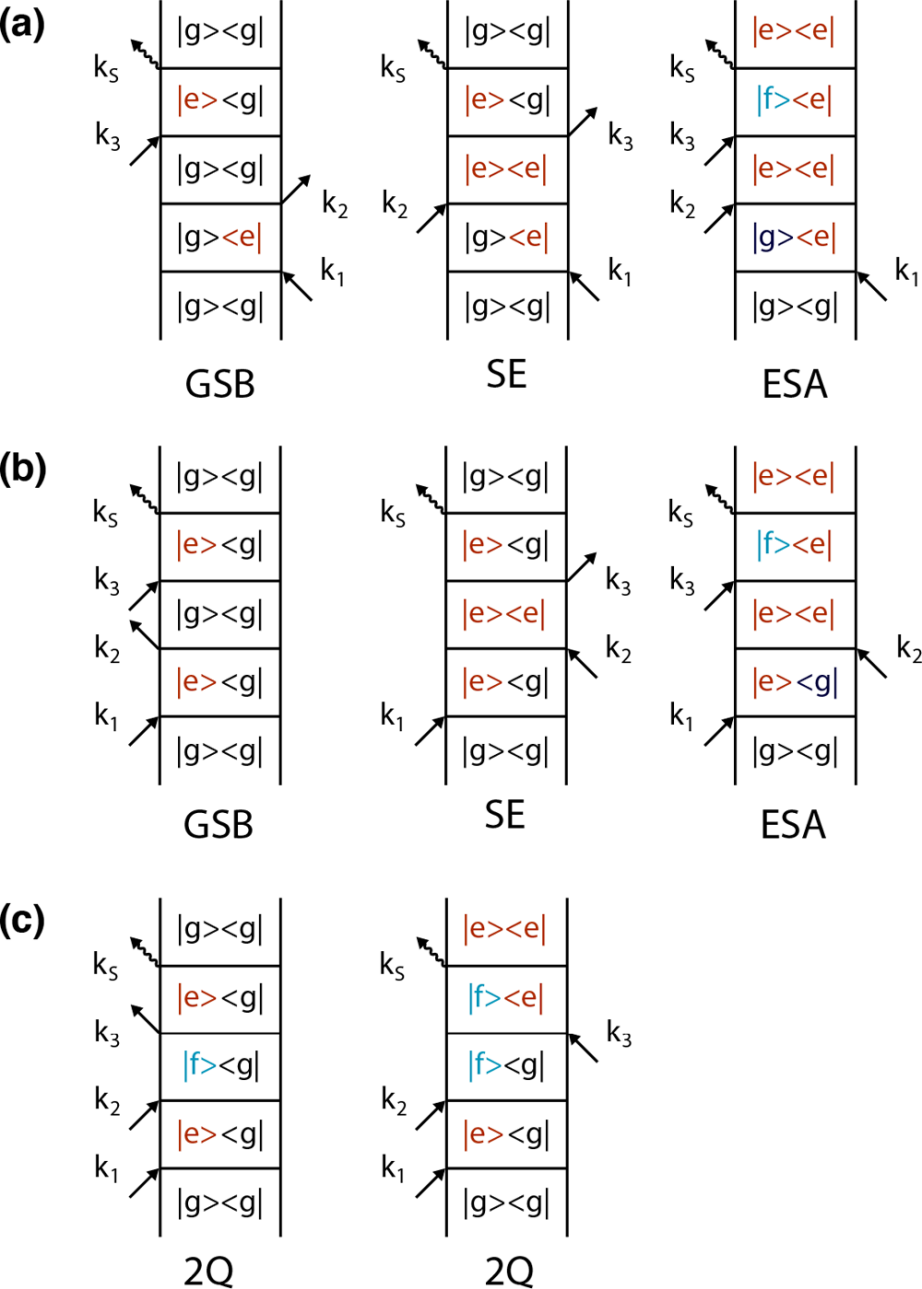
Feynman diagrams contributing to (a) rephasing, (b) nonrephasing, and (c) double-quantum signals.^32^ Orange is used to label single excited states, and green is used to label double excited states. GSB = ground state bleaching; SE = stimulated emission; ESA = excited state absorption. Conventionally, GSB and SE give positive contributions, while ESA gives negative contributions to the signal. For more details see refs (3, 25, 31, and 32).

In Figure 3, the Feynman diagrams most relevant for the interpretation of 2DES response are shown.

Each pathway corresponds to different pulse sequences and phase matching conditions, so that ***k***_sig_ = ± ***k***_1_ ± ***k***_2_ ± ***k***_3_ and **ϕ**_sig_ = ± **ϕ**_1_ ± **ϕ**_2_ ± **ϕ**_3_. In general, only a few pathways give a non-negligible contribution to the final signal and, based on that, rephasing (***k***_*R*_ = −***k***_1_+ ***k***_2_+ ***k***_3_), nonrephasing (***k*_*N*_**_*R*_ = +***k***_1_ – ***k***_2_ + ***k***_3_), and double-quantum (***k***_*2Q*_ = −***k***_1_ + ***k***_2_ + ***k***_3_) signals are typically defined.

A full treatment of third-order responses goes beyond the scope of this work, and we refer the interested reader to refs (3, 25, and 31) for an in-depth analysis. In practice, once relevant pathways/diagrams contributing to the response function are identified for the particular third-order experiment of interest, the total signal can be constructed by adding together the contributions from each diagram individually.

### 2.3 Diagonal and Off-Diagonal Signals

As exemplified in Figure 2, each diagram contributing to the total *R*^(3)^ gives rise to a signal at a specific (ω_1_, ω_3_) coordinate in the 2D map. However, typically, more than one diagram contributes to each relevant position, making the final interpretation of the peaks appearing in the 2D maps often tricky. Nonetheless, a few simplified guidelines can be drawn. The axis ω_1_ contains information about the first coherence excited just after the first interaction. It can be considered as a label of the initial excitation frequency. In contrast, the axis ω_3_ includes information on the optical coherence, which probes the state of the sample following the dynamics during the *t*_2_ period and the third interaction.

**Figure 4:**
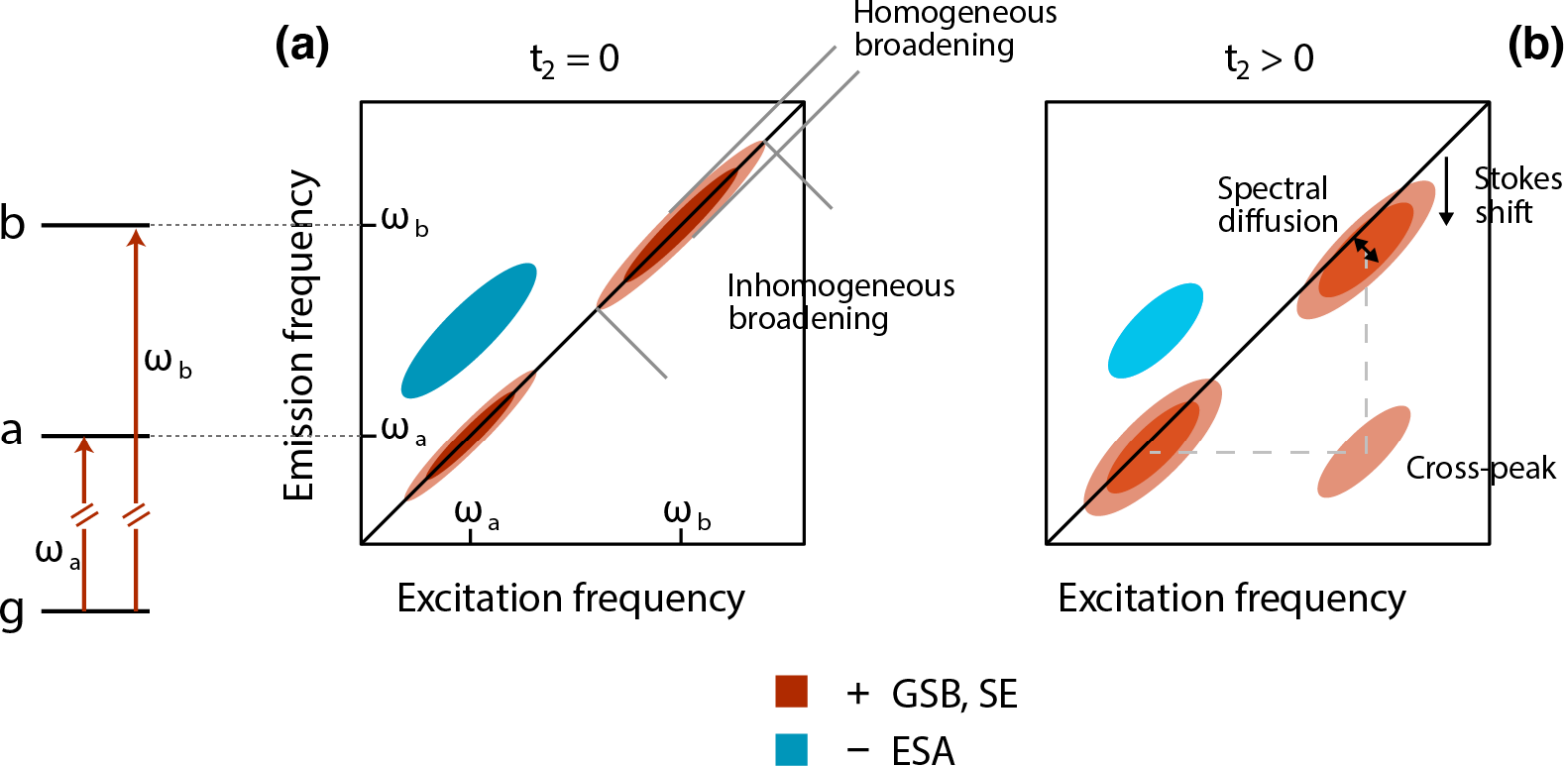
Schematic representation of 2D spectrum for a multilevel system. (a) At early population times, the GSB and SE features of two levels with frequency ω_a_ and ω_b_ appear as signals elongated on the diagonal. (b) As the population time evolves, the spectral diffusion produces a broadening of the features, the Stokes shift produces a redshift of the signals along the emission axis, and a cross-peak may appear in the lower part of the map as a consequence of the relaxation from the high energy level to the low energy one. Negative ESA signals can also appear.

By inspecting the corresponding Feynman diagrams, it is easy to demonstrate that signals appearing on the diagonal (ω_1_ = ω_3_) provide information on the excitonic structure of the system under investigation, i.e., on the frequency of the transitions falling in the experimental window. For example, in the rephasing map schematized in Figure 4a, two transitions with frequency ω_a_ and ω_b_, are found, suggesting the presence of two excited states, a and b.^24,25,29^

The intensity and shape of these bands change as the population of the two states decays to the ground electronic state. The shape of the peaks (particularly their diagonal and antidiagonal width) depends on the interactions with the environment and broadening mechanisms. Typically, as *t*_2_ increases, a broadening and rounding of the peaks is observed due to the loss of correlation (Figure 4b); this phenomenon is known as spectral diffusion, and the study of its time evolution is particularly relevant in the study of the configurational changes of the local environment and the time scales for the evolution of the bath.^33^ Relaxation within the same energy band during *t*_2_ may also manifest as a shift of the signals at lower emission frequency. Ultrafast Stokes shift due to the reorganization of the electronic clouds of the system and solvent appears as a drift of the features below the diagonal.^34^

**Figure 5:**
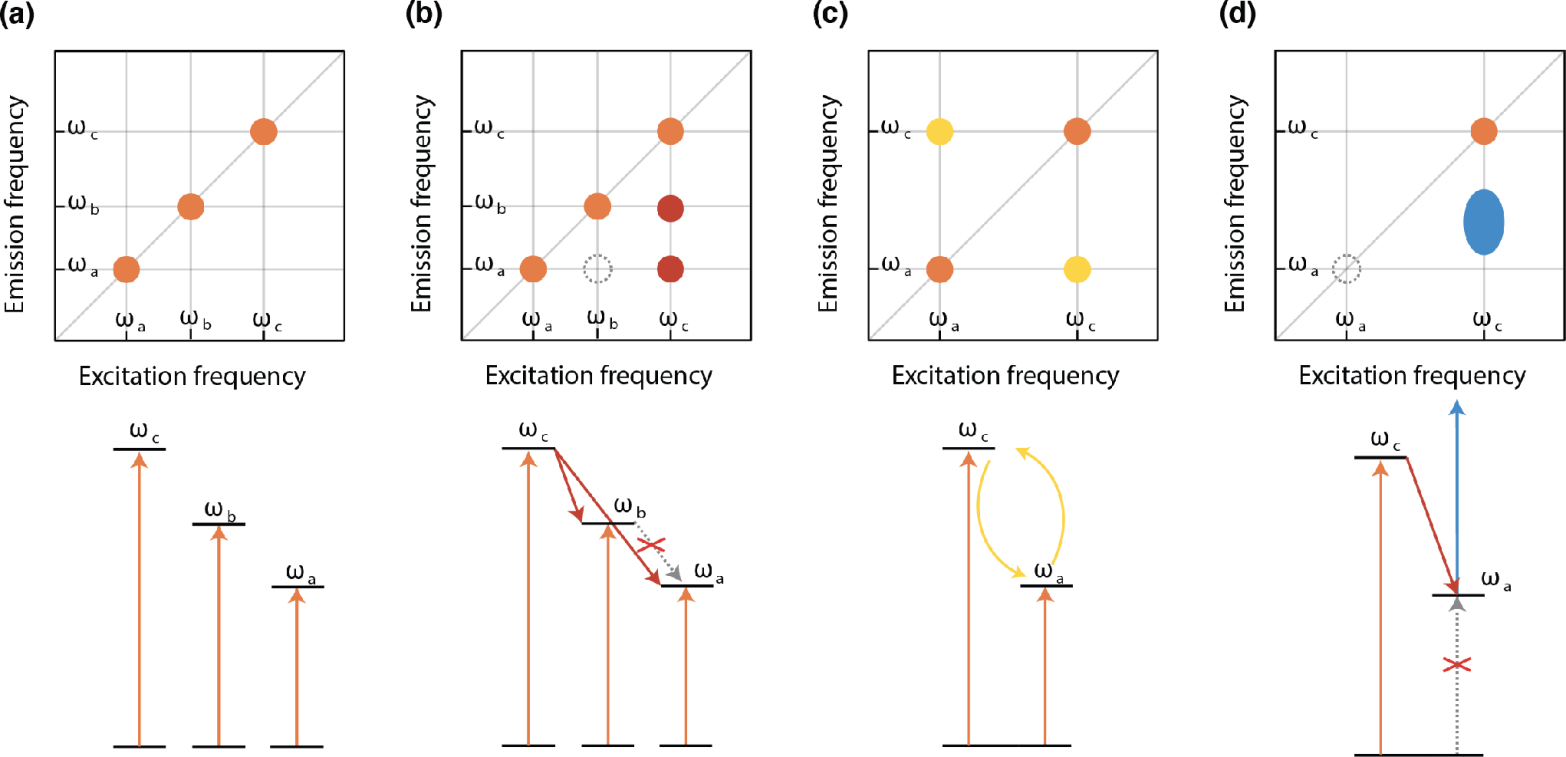
Position of the cross-peaks can be used to identify interaction pathways and their dynamics. (a) Isolated noninteracting two-level systems produce multiple diagonal peaks but no cross-peaks. (b) The cross-peaks at coordinates (ω_*c*_, ω_*b*_) and (ω_*c*_, ω_*a*_) appearing at *t*_2_ > 0 indicate that state *c* is coupled via energy transfer with states *a* and *b*. No coupling between states *a* and *b* because no cross peak appears at (ω_*b*_, ω_*a*_). (c) In the presence of resonance interactions between the two states, cross-peaks already appear at *t*_2_ = 0: the system can be modeled as a molecular (excitonic) dimer. (d) A dark state (ω_*a*_) can be characterized by the coupling with a bright state (ω_*c*_) in ESA processes.

A system that produces multiple diagonal peaks but no cross-peaks can be modeled as a set of isolated noninteracting two-level systems (Figure 5a). Relaxation between different energy levels, or energy transfer between different molecules, produces rising cross-peaks (ω_1_ ≠ ω_3_) below the diagonal with simultaneous decay of the diagonal signals associated with the initial states (Figure 5b). Excitation and emission coordinates of the cross-peaks directly provide the energy of the involved states. For example, excitation energy transfer or internal relaxation from one absorption band at energy ω_*a*_ to another at lower energy ω_*b*_ produces a cross-peak at (ω_1_ = ω_*a*_; ω_3_ = ω_*b*_), revealing the kinetics by which the ω_*a*_ state relaxes to the ω_*b*_ state. If the energy transfer is downhill in energy, then the cross-peak appears in the lower diagonal part of the 2D spectrum. Note that ω_*a*_ and ω_*b*_ can also be two vibronic states lying on the same electronic state. In this case, the cross-peak between ω_*a*_ and ω_*b*_ captures the vibrational relaxation process.

Cross-peaks can also arise in the presence of resonance interaction between two states, for example in a molecular dimer. In this case, the molecular states are no longer the eigenstates of the dimer, which are the delocalized excitations, termed excitonic states or excitons.^35^ The 2D spectrum of such a system (Figure 5c) contains cross-peaks arising from the coupling between the monomers, already at *t*_2_ = 0. Since they would not be present if the interaction between the molecular states was negligible, the presence of cross-peaks at very early times in 2D spectra implies coupling between the constituents of the observed system. More in general, cross-peaks will appear every time two levels are coupled and share a common ground state. The cross-peaks position can be used to identify which states are interacting, and the presence (or absence) of cross-peaks in a 2D map can provide insights into the nature of the electronic system.

This capability is also extended to dynamic processes involving dark states, which can be identified through the appearance of ESA cross-peaks. Indeed, dark states can be populated through relaxation from higher energy bright states, and once populated, an absorption from here to a higher excited state can be activated (Figure 5d). Conventional 1D femtosecond pump–probe experiments typically struggle to elucidate questions concerning the nature and significance of dark electronic states because their signals are overwhelmed by contributions from other strongly allowed transitions. As their name suggests, dark states cannot be caught through direct excitation because they have a null transition dipole moment. Nevertheless, the relaxation dynamics are strongly affected by the presence of such states, often involved in transfer, quenching, or photoprotection processes. An example is the leading role of the dark S_1_ state of carotenoids in light-harvesting complexes.^36,37^

In summary, one can say that taking advantage of the sensitive detection of coupling among states via cross-peaks attainable in 2DES, it is possible to detect not only the spectral signatures and kinetics of various (bright and dark) electronic states but also their interaction pathways.

### 2.4 Signal as a Function of *t*_2_: Population and Coherence Decay

**Figure 6:**
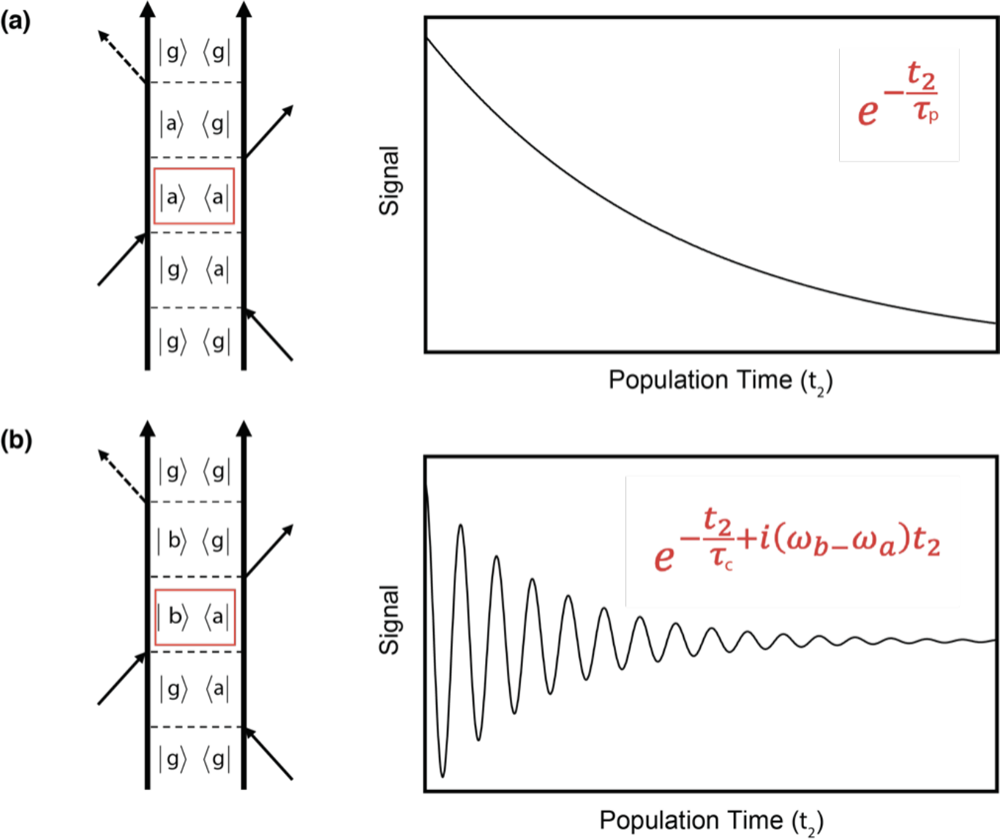
Examples of Feynman diagrams representing (a) nonoscillating and (b) oscillating contributions to the signal. The oscillation frequency corresponds to the difference between the frequencies of the states involved in the coherent superposition evolving during *t*_2_ (highlighted in red). Right panels sketch the dynamics of the corresponding signals as a function of the population time. τ_p_ and τ_c_ indicate the time constants for the population decay and coherence decay (dephasing), respectively.

We already discussed that in the response function formalism, the third-order signal could be expressed as a sum of contributions represented graphically by double-sided Feynman diagrams. These contributions can be classified into two groups depending on the signal’s evolution during *t*_2_, as shown in Figure 6. The first group includes nonoscillating pathways, represented by Feynman diagrams where the system reaches a pure state after the first two interactions (Figure 6a). The second group consists of oscillating contributions described by Feynman diagrams where, after the first two interactions, the system is in a coherent superposition of states (Figure 6b). In the first case, the signal evolves in *t*_2_ following the relaxation dynamics of the excited states that can be quantified through the solutions of suitable kinetic differential equations (“populations” dynamics). For example, in the simplest case of parallel relaxation processes, the solutions of the rate equations are real exponential functions.^38^ In the second case, the signal oscillates during *t*_2_ with a frequency proportional to the energy gap of the states that generate the coherence. These oscillations dampen over time according to their dephasing rates, depending on the nature of states themselves, on the temperature, on the environment, etc., and are well described by complex exponential functions.

The presence of these oscillations in the amplitude of the signal at specific coordinates is direct evidence for coherent dynamics, i.e., the time evolution of a coherent superposition of states. In other words, the capability of the technique to exploit the phase and coherence information in the time evolution of the optical polarization makes it sensitive to the presence of coherent mechanisms in the relaxation or energy transfer processes.

Depending on the character of the involved states, vibrational or electronic coherences can be distinguished. The amplitude patterns of the oscillations on the 2D spectra are different in the two cases and they can be used to identify the nature of the observed coherence^9,39^ Despite the significant differences of electronic and vibrational coherence signatures, due to the broadening of the observed features and laser spectrum distortions, it is not always easy to make a specific assignment. Furthermore, when the electronic coupling between chromophores generates mixed states the distinction becomes less rigorous, and a range of intermediate cases is possible.^40^

The identification of coherent beatings in the evolution of 2D maps, the analysis of their frequency and time behavior, and the ensuing interpretation of their possible electronic, vibronic, or vibrational nature is typically one of the essential steps in the analysis of the results of a 2DES experiment. Actually, it is precisely the sensitivity of 2DES to these beatings and the possibility of spreading their content information along three dimensions (ω_1_, ω_3_, *t*_2_) that makes this technique so powerful to detect signatures of coherent dynamics, especially in connection with energy, charge, or information transport.^41^

## 3 Implementations

One of the key requirements for coherent nonlinear spectroscopy is phase matching, which is achieved by properly adjusting the wavevectors **k**_***i***_ of the incident beams. Depending on the experimental geometry of the incident pulses, the phase-matching condition determines the direction of the signal emission. A range of geometries has been employed for 2DES measurements, including (i) fully noncollinear geometry in which every pulse has a different wavevector (2D photon echo, BOXCARS geometry), (ii) partially noncollinear pump–probe geometry in which the first two pulses are collinear and are followed by a probe pulse at a small angle (2D pump–probe), and (iii) fully collinear geometry in which every pulse has the same direction. All implementations have their advantages and disadvantages.

The fully noncollinear BOXCARS setup is probably the most frequently employed. In this implementation, the signal is generated in a background-free direction, and thus a pivotal advantage is a high signal-to-noise ratio. A fourth pulse, called the local oscillator (LO), is mixed with the signal in order to record the phase information recording the interference pattern of the two pulses. The main disadvantages of this implementation are the complexity of the setup, which must include in the design a passive phase stabilizer, and the fact that the final signal is accompanied by an arbitrary constant phase that must be determined.^23^

Collinear setups are intrinsically phase-stable because all the interacting pulses travel the same optical path. In these schemes, a pulse shaper is usually used to generate collinear pump pulses, with a known and adjustable relative phase. In the partially noncollinear pump–probe geometry, the probe beam can be either an attenuated replica of the pump or a spectrally broader white light continuum.^42^ The advantage of this scheme is that the signal is generated collinearly with the probe beam so that it is heterodyned with the probe itself and, therefore, automatically phased. The most relevant drawbacks are the strong background contribution, which lowers the sensitivity, and the difficulty of accessing the rephasing and nonrephasing portions of the signal separately in a straightforward manner. Indeed, the approaches so far successfully used to isolate the desired signal in a collinear geometry all rely on phase cycling^43,44^ or phase modulation^45,46^ and some form of lock-in detection. Both approaches are based on the principle that the signal phase depends on the phase of the excitation pulses. In phase cycling schemes, the phases of the excitation pulses are independently rotated by controlled amounts. Different spectra are recorded for any phase combination and then combined to remove any signals that do not depend on the phase of all three excitation pulses as forecasted by the response function theory. In a similar way, in phase modulation techniques, a continuous phase oscillation is applied to each of the beams. Therefore, the signal phase will be modulated at different frequencies corresponding to specific linear combinations of the three frequencies used to modulate the phase of the input beams. Signals corresponding to different pathways (for example the rephasing and nonrephasing signals) will be modulated at different frequencies. The rotation or modulation of the phases of the exciting pulses is typically performed by exploiting the same pulse shaper apparatus used to generate the pulse sequence. Thus, additional equipment is not required.

While in its first applications the BOXCARS noncollinear geometry was preferred, lately, collinear setups are quickly gaining ground because of the possibility to switch to the so-called “action detected” techniques.^45−48^ In these configurations, a fourth pulse is added to the pulse sequence to drive the system into an excited or ground state population. In phase modulation or phase cycling schemes, the excited state population is also modulated, and then it can be read out by some other means, for example, photocurrent or photoluminescence. These techniques are particularly inspiring in view of characterizing operating devices or realizing *ad hoc* schemes to answer specific questions (see Section 4).

## 4 Characterization of Complex Dynamics toward Quantum Technology Applications

2DES, especially in the first decade after its practical development, has been often associated with the investigation of energy and charge transfer dynamics in biological photosynthetic processes. Since the first examples of investigations on the excitonic structure and dynamics in the Fenna–Mathew–Olson (FMO) complex,^9,49,50^ 2DES has been applied to many systems and problems in photosynthesis research. For instance it was used to uncover the mechanisms of charge separation in Photosystem II, Photosystem I, and the bacterial reaction centers and to untangle the intricate pathways in the light-harvesting antennas of green photosynthetic bacteria, purple bacteria, cryptophyte algae, brown algae, and green plants.^6^ Variants of the technique have also been developed to follow the excitation energy flow in intact living cells of photosynthetic organisms.^51^

A lot of attention was also paid to studying the dynamics of isolated biologically relevant chromophores in vitro. For example, the ultrafast relaxation dynamics in the sub 100 fs time regime of chlorophyll *a*,^52,53^ chlorophyll *b*,^54^ bacteriochlorophyll *a*,^55^ and carotenes^56−58^ have been elucidated through 2DES. A thorough characterization of these dynamics is crucial for a complete understanding of the mechanisms regulating the ultrafast dynamics of the relaxation processes in the more complex multichromophore light-harvesting systems in which these molecules are embedded.

Several of these studies have been devoted to analyzing the role of quantum coherences in the photosynthetic processes. Despite two decades of investigations, this remains a highly engaging albeit controversial issue.^9,59,60^ There is, however, a general consensus in admitting that 2DES played an essential role in resolving the dynamics and pathways of energy and electron transport in various light-harvesting antenna systems and reaction centers with an unsurpassed level of detail.

The investigation on the role of quantum effects in the dynamics of photosynthetic complexes, regardless of whether or not these effects are relevant in nature, had the merit to trigger important new lines of research. Indeed, it is questionable whether quantum effects, even if nature does not exploit them, can be engineered into artificial systems designed *ad hoc* to control quantum coherent energy or charge transfer.^61^

In this sense, 2DES played a crucial role in inspiring new technologies and materials where quantum coherence is used as a new foundational principle to realize devices with improved performances.^62^ The challenge is now moving from fundamental studies to actual technology, and this requires the development of suitable materials where quantum phenomena are sufficiently controllable to be exploitable. Despite the tremendous potentiality of quantum technologies, their concrete demonstration in real devices is still limited to few niche applications.^62^ In a very simplistic way, this goes back to the complexity of quantum mechanics principles and, even more, to the difficulty of maintaining quantum features active in our macroscopic world, intrinsically classic. It was suggested that our world is “too wet, hot, and noisy” to preserve the phase and amplitude of the quantum mechanical coherent superpositions. And so too, our devices.^7^ Indeed, energy and charge transfer dynamics are inevitably affected by the thermal fluctuations of nuclear motions. This is because the magnitude of the fluctuations in site energy and electronic coupling can be comparable with the magnitude of the electronic coupling that causes excitation energy and charge transfer.

Therefore, the challenge of achieving control over quantum phenomena must necessarily pass through the microscopic understanding of the coupling among the photoactive electronic systems and between these systems and the environmental bath. And, as emerging from the previous sections, 2DES appears as the ideal tool to characterize such couplings and face this challenge. A few relevant recent examples of how 2DES can effectively be employed in this context are collected in the following sections.

### 4.1 Artificial Molecular Nanosystems

The role of the coupling system-environment is now emerging as one of the key factors to be controlled (and engineered) to be able to fully exploit the quantum nature of the transport processes. Photosynthetic protein complexes represent one of the best examples of how nature can tune the electronic properties of chromophores, their interactions and relaxation and transport dynamics by embedding such chromophores in a suitably “engineered” environment, i.e., the protein scaffold.^63^ Inspired by that, several artificial biomimetic multichromophore systems have been proposed, where the photoactive chromophores were embedded into “structured environments” by covalent linking or by supramolecular self-assembly techniques. Examples of this approach are dimers of interacting chromophores mounted on DNA strands,^64,65^ chromophores covalently attached to polymeric chains,^66^ self-assembled aggregates of dye-functionalized short amino acid sequences,^67^ porphyrin nanorings^68^ and J-aggregates,^10,11^ and H-bonded dimers.^69^

**Figure 7:**
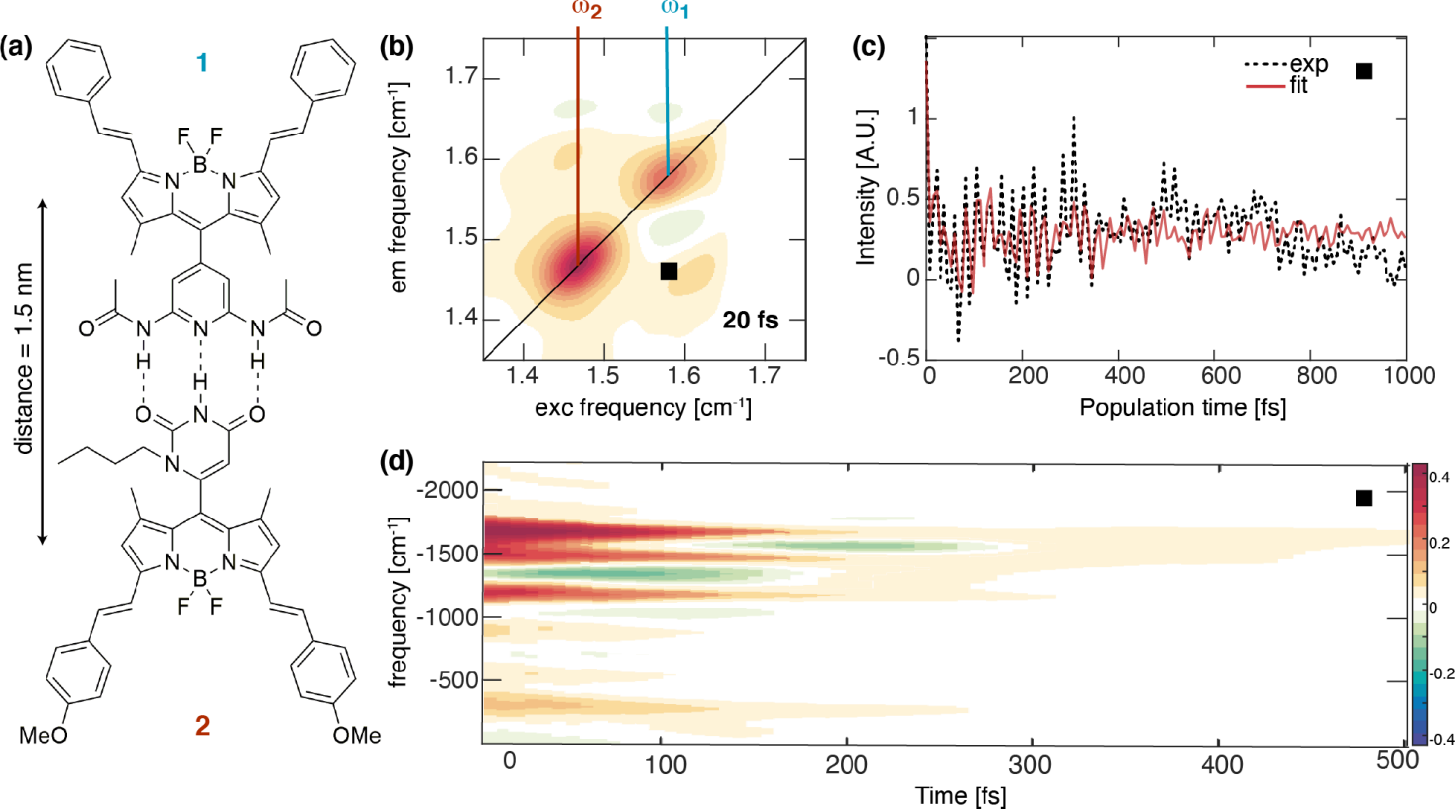
Effect of H-bonds on the ultrafast dynamics of an H-bonded dimer of BODIPY molecules.^69^ (a) Molecular structure of the dimer. (b) Purely absorptive 2DES map of the dimer collected at *t*_2_ = 20 fs. The transition energies of the two molecular moieties are highlighted in red and blue. (c) Signal decay as a function of *t*_2_, extracted at the cross-peak coordinates pinpointed by the square in panel b. (d) Time–frequency transform^76^ of the decay trace in panel c showing the frequency (*y*-axis) and the time behavior (*x*-axis) of the main beating components, corresponding to vibrational motions of the H donor and acceptor groups.

This growing amount of evidence is now permitting us to extract critical structure-to-property relationships to design new materials where the control of at least a few relevant aspects of the system-environment coupling leads to control of coherent dynamics. For example, refs (69−71) suggest that the establishment of specific and directional interactions like hydrogen bonds (H-bonds), can have very strong consequences for the electronic coupling and the ultrafast dynamics of coupled chromophores. In particular, new intra- and intermolecular ultrafast relaxation channels can be activated, mediated by the vibrational motions of the hydrogen donor and acceptor groups, also when the coupled chromophores are at significant distances (Figure 7). These findings suggest that the design of H-bonded structures is a particularly powerful tool to drive the ultrafast dynamics in complex materials. The intrinsic multidimensionality of the 2DES technique, its capability of resolving dynamic pathways in frequency and in time, and the sensitivity to couplings mapped at cross-peak positions have been absolutely essential in achieving such a level of understanding of the complex mechanism regulating such dynamics.

Another significant emerging aspect deals with the flexibility of the scaffold and the different degree of conformational disorder.^66,72^ It has been verified that conformational disorder affects predominantly low-frequency vibrational modes, delocalized on the chromophores and the scaffold. The role of low-frequency vibrational modes in the coherent dynamics of complex systems represents a nodal issue in identifying mechanisms capable of preserving electronic coherence in biological, organic, and inorganic assemblies.^73−75^ The decoherence dynamics promoted by conformational disorder are thus emerging as an essential ingredient to describe the dynamics and properties of multichromophore compounds.

### 4.2 Strong Light–Matter Interactions: Molecular Polaritons

The coupling between the photoactive electronic system and its environment plays a decisive role in the excited state dynamics of biological and artificial molecular systems. To achieve control over the coupling between the system and the thermal bath in condensed matter is generally a difficult challenge. Nonetheless, a recently proposed approach highly promising for technological application is the exploitation of strong light–matter coupling. When a strong coupling between an electromagnetic field and molecular moieties is established, hybrid states, called polaritons, are formed, with mixed molecular and photonic character. In this regime, both photons and molecular excited states lose their individuality. The generic setup to build polaritonic excitations consists of a nanostructure able to confine the electromagnetic fields to the microscale and a molecular moiety with one or more transitions nearly resonant with the electromagnetic modes of the nanomaterial.^77^ Typically, this corresponds to molecules embedded into optical microcavities or assembled on the surface of a metal nanostructure supporting plasmons.

Theoretical predictions suggested that the system–bath coupling properties can be controlled using the polaron decoupling effect, in which a coherent interaction between molecular excitons and photons reduces the reorganization energy, leading to significant changes in the rates of photophysical processes.^78^ These theoretical predictions have been recently experimentally demonstrated by 2DES applied to a periflanthene derivative film in a microcavity.^18^ It was suggested that the strong light–matter coupling established in this system suppresses the dephasing of electronic coherences, possibly supporting coherence-assisted processes. This approach appears highly promising toward the development of quantum technologies devices where the promotion of hybrid polariton states can control the dephasing of quantum coherence. It is expected that 2DES measurements will play an essential role in achieving a better understanding of the physical and dynamic phenomena at the base of this still underexplored but encouraging effect.^19^

### 4.3 Colloidal Semiconductor Nanocrystals (Quantum Dots)

Semiconductor nanocrystals (quantum dots, QDs) have attracted vast interest given their peculiar size-dependent optical and electronic properties. Since the early 1990s, colloidal synthesis has opened extraordinary possibilities for tuning their optical and electronic properties by controlling size, shape, and crystallographic structure. The applications of these colloidal nanostructures range from optoelectronics, including photovoltaics, diodes, and photodetectors, to bioimaging and photocatalysis.^79^ Although the technological exploitation of QDs is an already mature field, several even more promising additional applications can be envisioned, mainly connected with the possibility of exploiting their ultrafast (coherent) photophysics, which represents still an under-explored field. 2DES spectroscopy appears ideal to this aim, but it is only recently that it has been effectively applied to QDs.^80^

For example, ref (13) represents an excellent example of how it is possible to benefit from the combined use of different 2DES experimental schemes to achieve a comprehensive understanding of the ultrafast relaxation phenomena in QDs samples. A fully noncollinear BOXCARS setup has been selected for its better time resolution to access the subpicosecond dynamics of hot excitons cooling, whereas a partially collinear 2D pump–probe setup granted access to multiexciton relaxations, whose characterization requires higher fluence and longer time windows. These measurements provided a unique global visualization of the sample dynamics in view of application to novel and innovative nanomaterials.

A particularly debated topic is the possibility of recording coherent beating in the 2D signal amplitude of QDs samples. The capability to exploit coherent quantum phenomena in nanometer scale materials is at the forefront of the most recent quantum technology applications. However, the development of this technology is strongly dependent on a deep understanding of how to generate, manipulate, and characterize a coherent superposition of quantum states in the nanosystems.

In view of using QDs as candidates for quantum technology devices, the time evolution of coherence superpositions of electronic levels is of fundamental interest not only to understand the mechanisms of dephasing but also to harness the quantum nature of the coherent phenomena in devices.

Although the first investigations on the ultrafast coherent dynamics in semiconductors date back to the 1980s,^81^ 2DES could really provide remarkable new insights into this topic. Despite the noteworthy recent advancements in data acquisition and analysis techniques, the experimental reports about coherent superpositions of different excitonic bands in QD samples remain limited,^16,82−87^ and the debate about their effective experimental detection is still open.^80,87^

The main reasons that complicate the detection of coherent dynamics in QDs samples are (i) the inhomogeneous broadening, intrinsically affecting QDs samples and resulting in a quick ensemble dephasing (“fake decoherence”)^88^ and (ii) the contribution of the nonresonant response of the solvent affecting the dynamics at short time delays (coherent artifact) and also contributing at longer times through impulsive Raman modes.^13,80^ Nonetheless, it was recently found that these two hindering effects can be overcome through a suitable choice of size dimensions, ligands, and solvent.^14,21^ The demonstration that coherent dynamics can emerge even from a sizable inhomogeneous ensemble is a particularly relevant issue in view of quantum technology applications and coherent control.^89^

### 4.4 Solid-State Materials and Operating Devices

The up-to-date proposals for the realization of QD-based quantum devices are mainly using very low temperatures or room temperature isolated units, where a single excitation per dot is considered, to minimize decoherence effects.^89^ However, the possibility of assembling networks of coherently interacting dots at ambient conditions, especially in the solid-state, is particularly interesting for the possibility of large-scale integration and the development of complex connectivity. Indeed, in the context of optical information processing in solids, establishing controlled channels of coupling within an ensemble of isolated units represents a truly challenging but highly rewarding goal.

To this aim, solid-state assemblies of colloidally grown QDs^90^ appear highly promising. In solid-state assemblies of colloidal QDs, depending on size dimensions and interdot distances, the coupling between different QD units (*inter*dot coupling) ranges from long distance dipole–dipole interactions to short-range exchange interaction, the last involving delocalization of wave functions over two or more dots. The collective nature of excitations in these strongly interacting samples can be ascertained by the presence of red-shifted bands in the absorption and photoluminescence spectra^91,92^ and improved charge transport,^90^ as a result of coherent delocalization. However, the effective exploitation of strongly interacting QDs networks envisioned as fundamental units of quantum circuits also requires the dynamical characterization of these collective quantum mechanically coupled states in the ultrafast time scale, although the challenging nature of these measurements hindered for a long time a thorough investigation of these dynamics.^88^

**Figure 8:**
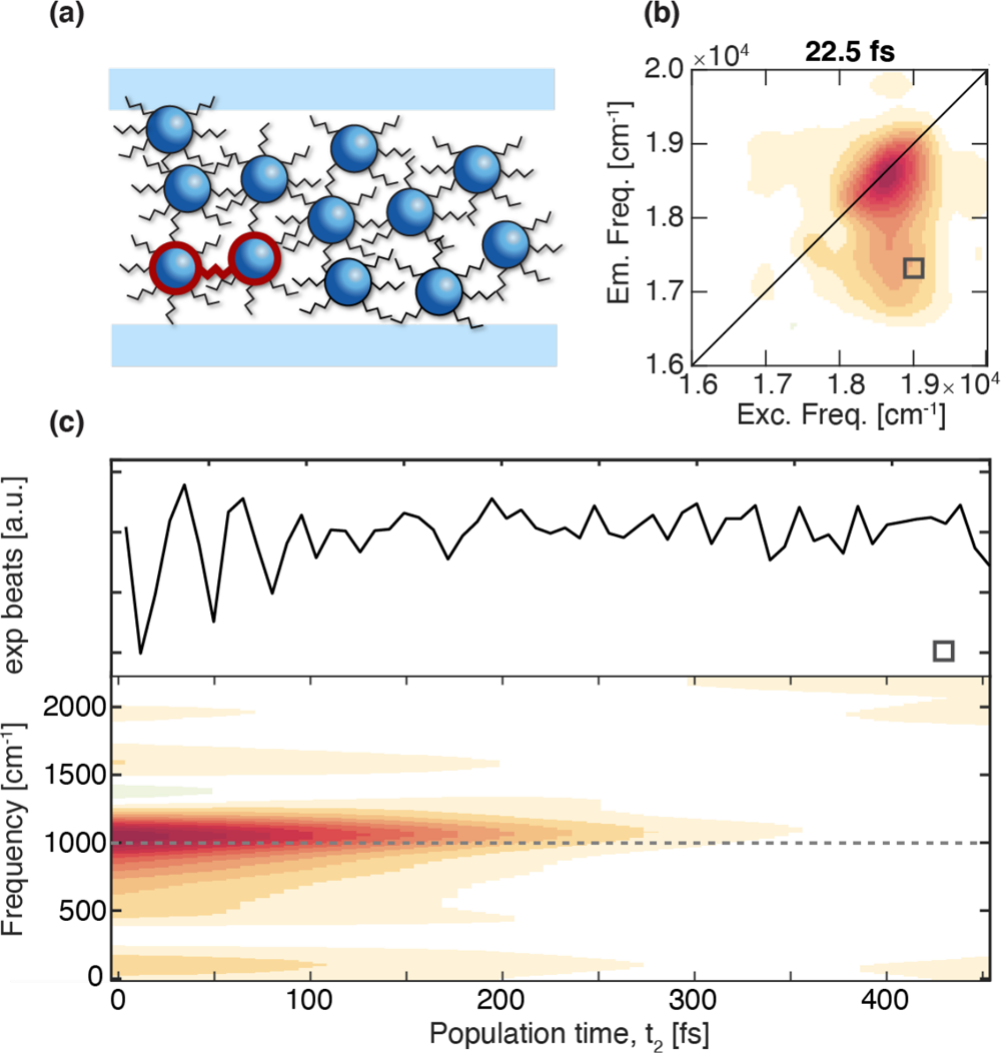
Characterization of interdot coherence in disordered solid-state multilayer QDs films. (a) Pictorial representation of the sample. (b) Purely absorptive 2DES map of the film collected at *t*_2_ = 22.5 fs. (c) Oscillating residues as a function of *t*_2_, extracted at the cross-peak coordinates pinpointed by the square in panel b (upper line), and corresponding analysis in terms of time-frequency transform^76^ (lower line).

In refs (21, and 93), 2DES has been applied to solid-state QDs materials, prepared using a self-assembly method to create a disordered layered structure of QDs, coupled by covalently bonded organic molecules (Figure 8). Preliminary measurements by pump–probe spectroscopy captured the presence of energy transfer rates among QDs compatible with coherent mechanisms of energy transfer.^94^ Nonetheless, it was only by applying 2DES that it was possible to directly detect the dynamic evolution of a coherent superposition of states delocalized over more than one QD. Supported by theoretical simulations and through the comparison with the response of noninteracting samples, 2DES provided solid evidence of interdot coherences in such solid-state materials. Even more interesting, both theoretical and experimental results seem to indicate that the electronic coherences between excitons delocalized over the two dots have rather long dephasing times (100–200 fs), likely connected to the specific properties of these states.^93^ Although additional investigations are needed, these findings are opening new avenues for the effective exploitation of these materials for quantum technologies purposes, also providing important guidelines for the choice of future materials to be employed in QD-based devices.

Also in this case, the possibility of spreading the signal along two dimensions facilitated the clear identification of interdot coherent features among the other intradot dynamics, and this elected the 2DES technique as one of the most informative tools at our disposal for the screening of materials for quantum technologies.

The application of 2DES to solid-state samples poses however several challenges. In the 2DES investigations summarized in Figure 8a–c, the solid state samples were thick isotropic disordered systems, assimilable to “solid-state solutions”. However, for future quantum technology applications, the use of ordered microscale samples or heterogeneous materials with localized μm structural domains can be envisioned. For these samples, the standard fully noncollinear geometry cannot be employed because the necessary phase-matching condition can be achieved only with longer focal lengths, leading to larger spot sizes (on the order of hundreds of micrometers) and averaging over different spatial regions.^95^ Moreover, this configuration also typically requires samples with a considerable thickness, a condition not always fulfillable in solid-state devices.

To overcome this limitation, one of the latest and most promising developments of 2DES is the possibility to replace the coherent optical detection of a third-order signal with the detection of a signal directly generated by an excited state population stimulated by a four-pulse sequence. As described in Section 3, these new action-detected configurations are based on fully collinear geometries, which permit the relaxation of the phase-matching conditions.^47^

The first advantage of the collinear alternative is that high-numerical-aperture objectives can be implemented in place of long-length focusing optics. In this way, all incident light arrives at the sample from the same solid angle reducing the interaction volume and leading to spatial resolution on the order of hundreds of nanometers. On the one hand, this permits us to improve 2DES by adding spatial resolution, crucial for investigating solid-state devices. On the other hand, this points toward the exciting possibility of performing coherent multidimensional spectroscopy at the single molecule level. In this context, local microscopic 2DES using fluorescence detection has already been demonstrated,^96^ and applied for example to single-layer transition metal dichalcogenides MoSe_2_ samples, a promising platform for new photonic, optoelectronic, and quantum devices.^97^

Interestingly, it is expected that this spatially resolved 2DES approach will be extended to other relevant device materials, such as layered heterostructures, perovskites, bulk heterojunctions, single-wall carbon nanotubes, microcavities, and so on.^22^

Moreover, this configuration intrinsically calls for the combination of 2DES with other spectroscopic techniques. Indeed, the detection is not limited to the nonlinear polarization but potentially extends to any kind of observable proportional to population conditions after the fourth pulse interaction, like photocurrent, photoelectron emission, or mass spectroscopy. In 2014 Karki et al.^20^ applied photocurrent-detected 2DES to a PbS quantum dot photocell. Subpicosecond evolution consistent with multiple exciton generation has been found. Since the measurement is based on detecting the photocell current *in situ*, the method is particularly suited to study the fundamental ultrafast processes that affect the function of the device. This opens new avenues to investigate and implement coherent optimization strategies directly within working devices. Reference (98) discusses all the aspects that make photocurrent-detected 2DES a technique of choice where the device’s photophysics is concerned.

## 5 Concluding Remarks

More than 20 years have passed since the first experimental realizations of 2DES. In these first pioneering experiments, the attention was focused mainly on the characterization of the mechanisms and dynamics of the energy migration during light-harvesting in biological photosynthetic antenna complexes. The sensitivity of the technique to interchromophore couplings, its capability of identifying with unprecedented clarity transport processes, and coherent dynamics have been crucial for the blossoming of the quantum biology discipline. Twenty years later, we forecast that 2DES will also have a similar role in the emerging field of quantum technology. Novel experimental schemes and increasingly sophisticated data analysis methodologies are being proposed nowadays, capable of overcoming most of the drawbacks of the standard 2DES techniques when applied to solid-state materials or (nano)devices for quantum technology applications. In this context, the new implementations based on fully colinear setups appear particularly appealing. First, the possibility of moving from the detection of a coherent field to a detection extended to any kind of observable proportional to the final population conditions (fluorescence, photocurrent, photoelectrons, etc.) holds great promise to expand the range of physical problems tackled by 2DES. Second, the entire experiment can be managed only through the use of a pulse shaper apparatus, with no need for complex optical layouts. In this way, more compact and simplified setups can be foreseen, which will ease the transition of 2DES from a niche technique limited to highly specialized laboratories to a more user-friendly experiment. This is a crucial step to move 2DES toward the technological transition envisioned in quantum technology applications. Moreover, the same pulse shaper apparatus can be programmed to generate several different pulse sequences, including also a higher number of pulses and different phase matching conditions. The high flexibility of this approach is very promising in view of designing new experimental schemes to address specific problems arising in the characterization of new materials.

This transition is partly already begun, and indeed, some of the more recent results highlighted and discussed in this Perspective are already testifying to the enormous potential and versatility of the 2DES techniques to impact the field of nanosystems, semiconductors, quantum technologies, and quantum devices.
